# Mini-Konno procedure for aortic stenosis with small aortic annulus

**DOI:** 10.1016/j.xjtc.2025.09.030

**Published:** 2025-10-13

**Authors:** Daisuke Yoshioka, Ai Kawamura, Takuji Kawamura, Shin Yajima, Yusuke Misumi, Shunsuke Saito, Kazuo Shimamura, Shigeru Miyagawa

**Affiliations:** Cardiovascular Surgery, Osaka University Graduate School of Medicine, Suita, Osaka, Japan

**Figure:**
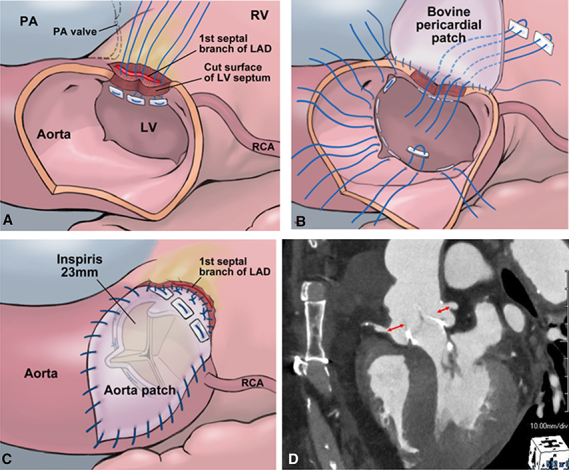
Mini-Konno enables annular and sinus enlargement, ensuring adequate VTA and VTC.

**Central Message**

Mini-Konno with RV preservation allowed prosthetic valve implantation and sinus/LVOT enlargement, ensuring future valve-in-valve feasibility through maintained anatomical configuration.

With the growing recognition of the importance of lifetime management of patients with aortic stenosis, annular enlargement at the initial aortic valve replacement has gained importance. Several annular-enlargement techniques have been described, including the Y-incision and Manouguian methods,^1^^,^^2^ whereas the Konno procedure is most commonly performed in pediatric patients.^3^ In adult cases in which the Konno procedure has been applied, it has been reported that the valve-to-aorta (VTA) and valve-to-coronary (VTC) distance are better preserved compared with the Y-incision technique, rendering it more suitable for future valve-in-valve procedures.^4^ However, the Konno procedure requires incisions in both the right ventricular (RV) free wall and the interventricular septum, raising concerns regarding postoperative RV dysfunction. Herein, we describe the Mini-Konno technique, which allows both preserving RV function and annular enlargement with feasible future valve-in-valve procedures.

## Case Report

Written informed consent for publication was given from the patient about this report (institutional review board number 08218-12, approval April 10, 2025). A 76-year-old man with body surface area of 1.79 m^2^ presented with exertional dyspnea and was diagnosed with severe aortic stenosis. Echocardiography showed a left ventricular dimension of 53/30 mm and an ejection fraction of 72%, with a mean gradient of 56 mm Hg. Contrast-enhanced computed tomography revealed an annular diameter of 21.9 mm and a sinotubular junction of 24.9 mm. The predicted indexed effective orifice area with a 21-mm valve was 0.76 cm^2^/m^2^, indicating moderate prosthesis-patient mismatch (PPM). Therefore, a 23-mm INSPIRIS RESILIA valve was considered necessary to avoid PPM.

## Surgical Technique

The surgical technique is demonstrated in Video 1. After the crosscramp, the aorta was obliquely incised to the right coronary cusp/left coronary cusp commissure. The calcified valve and annulus were excised. A 21-mm sizer passed tightly, but the 23-mm sizer could not, confirming the need for annular enlargement. The outside of the aortic root was dissected downward toward the ventricular muscle. The ventricular septal muscle was carefully divided into the left ventricular (LV) and RV septal components, and dissection continued until the first septal branch of the left anterior descending coronary artery was identified transversely. The aortotomy incision was extended to the annulus and the divided LV septal muscle until the first septal branch.

**Figure 1:**
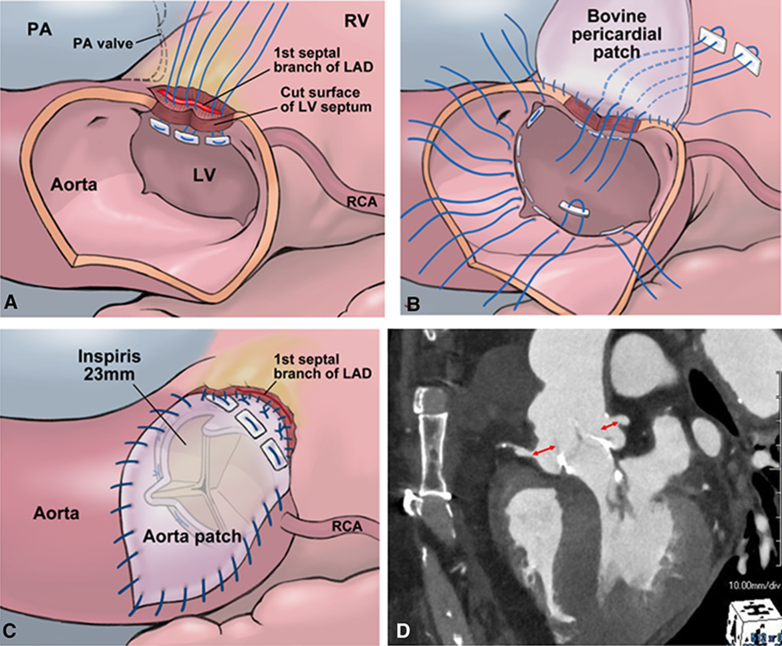
Operative steps of the Mini-Konno procedure, showing septal incision and patch reconstruction (A), suture placement for aortic valve replacement with annular enlargement (B), aortotomy closure with bovine pericardial patch (C), and postoperative CT demonstrating enlarged sinus with preserved valve-to-aorta and valve-to-coronary distances (D). *PA*, Pulmonary artery; *RV*, right ventricle; *LAD*, left anterior descending; *LV*, left ventricle; *RCA*, right coronary artery.

Three 4-0 monofilament U-shaped sutures with pledgets were placed from the inside to the outside of the incised LV septum (Figure 1, *A*). These sutures were passed through an autologous pericardial strip for reinforcement and a bovine pericardial patch, which was trimmed to reconstruct both the LV septum and the aortic annulus. The sutures were tied, followed by a second running suture extending from the base of the bovine pericardial patch to the aortic annulus and Valsalva sinus.

Sutures for aortic valve replacement were placed. In the region of the annulus reconstructed with the bovine pericardial patch and its adjacent areas, 3 mattress sutures were passed from the outside to the inside (Figure 1, *B*). Although valve sizing demonstrated that a 25-mm prosthesis could pass through, a 23-mm prosthesis was selected in order to preserve the VTA and VTC distances. In total, 15 sutures were placed in the neoannulus, and a 23-mm INSPIRIS RESILIA valve was positioned supra-annularly.

The aortotomy was closed after trimming the remaining portion of the bovine pericardial patch (Figure 1, *C*). Before releasing the crossclamp, fibrin glue was applied to seal the interface between the reconstructed LV patch and the dissected RV in order to prevent bleeding. The crossclamp was released with spontaneous heartbeat recovery and no atrioventricular block. Bleeding was minimal, and the patient was weaned uneventfully from cardiopulmonary bypass.

The procedure was completed with crossclamp and cardiopulmonary bypass times of 86 and 126 minutes, respectively. The postoperative course was uneventful, and the patient was discharged on postoperative day 15. The prosthetic valve functioned well with a mean gradient of 7 mm Hg, and its effective orifice area index was 1.25 cm^2^/m^2^. Valsalva sinus enlargement ensured adequate VTA and VTC distance (8 mm to right, 7 mm to left coronary orifice) for future valve-in-valve (Figure 1, *D*).

## Comment

In this case, annular enlargement was achieved using the Mini-Konno technique, permitting adequate left ventricular outflow tract enlargement and implantation of an appropriately sized prosthetic valve, thereby avoiding PPM. A key advantage of this technique is preservation of the RV myocardium, potentially reducing the postoperative RV dysfunction risk. In "acc3addition unlike the Y-incision and Manouguian techniques, the Mini-Konno involves patch augmentation between the right and left coronary cusps, increasing the distance between the prosthetic valve and the coronary ostia. This anatomical configuration may reduce the risk of coronary obstruction during future transcatheter valve-in-valve procedures.

Several technical considerations must be noted. The anatomical location of the first septal branch varies; in some cases, limited resection may compromise annular enlargement and adequate valve upsizing. Moreover, although the conventional Konno carries a risk of residual shunt and RV dysfunction, this Mini-Konno may involve bleeding from the left ventricular septum.

## Conflict of Interest Statement

The authors reported no conflicts of interest.

The *Journal* policy requires editors and reviewers to disclose conflicts of interest and to decline handling or reviewing manuscripts for which they may have a conflict of interest. The editors and reviewers of this article have no conflicts of interest.

## Supplementary Data

Video 1 Movie of Mini-Konno surgery. Video available at: https://www.jtcvs.org/article/S2666-2507(25)00452-3/fulltext.
