# Supplementary figures and images for: Mini-Konno procedure for aortic stenosis with small aortic annulus
Source: JTCVS Tech. 2025 Oct 13;35:102117. doi: 10.1016/j.xjtc.2025.09.030 (PMC12881728; doi:10.1016/j.xjtc.2025.09.030)

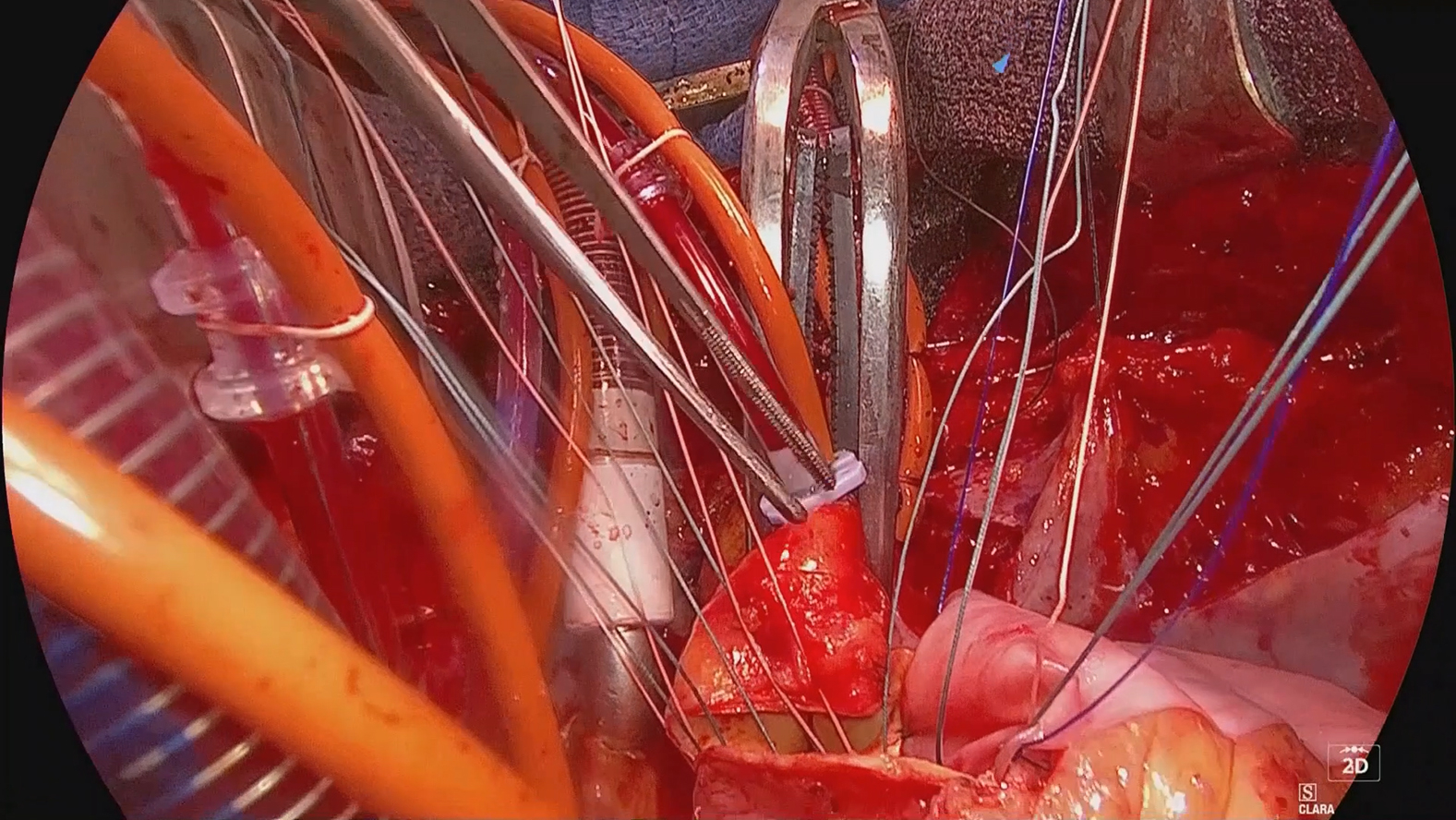

Supplement: Video 1 — Movie of Mini-Konno surgery. Video available at: https://www.jtcvs.org/article/S2666-2507(25)00452-3/fulltext. [file fx2.jpg]
